# New interleukin-15 superagonist (IL-15SA) significantly enhances graft-versus-tumor activity

**DOI:** 10.18632/oncotarget.17875

**Published:** 2017-05-15

**Authors:** Cavan P. Bailey, Tulin Budak-Alpdogan, Christopher T. Sauter, Michelle M. Panis, Cihangir Buyukgoz, Emily K. Jeng, Hing C. Wong, Neal Flomenberg, Onder Alpdogan

**Affiliations:** ^1^ Department of Medical Oncology, Kimmel Cancer Center, Thomas Jefferson University, Philadelphia, PA, USA; ^2^ Altor BioScience Corporation, Miramar, FL, USA; ^3^ Department of Hematology and Oncology, MD Anderson Cancer Center at Cooper, Camden, NJ, USA

**Keywords:** stem cell transplantation, interleukin-15, cytokine therapy, graft-versus-tumor activity, animal models

## Abstract

Interleukin-15 (IL-15) is a potent cytokine that increases CD8^+^ T and NK cell numbers and function in experimental models. However, obstacles remain in using IL-15 therapeutically, specifically its low potency and short *in vivo* half-life. To help overcome this, a new IL-15 superagonist complex comprised of an IL-15N72D mutation and IL-15RαSu/Fc fusion (IL-15SA, also known as ALT-803) was developed. IL-15SA exhibits a significantly longer serum half-life and increased *in vivo* activity against various tumors. Herein, we evaluated the effects of IL-15SA in recipients of allogeneic hematopoietic stem cell transplantation. Weekly administration of IL-15SA to transplant recipients significantly increased the number of CD8^+^ T cells (specifically CD44^+^ memory/activated phenotype) and NK cells. Intracellular IFN-γ and TNF-α secretion by CD8^+^ T cells increased in the IL-15SA-treated group. IL-15SA also upregulated NKG2D expression on CD8^+^ T cells. Moreover, IL-15SA enhanced proliferation and cytokine secretion of adoptively transferred CFSE-labeled T cells in syngeneic and allogeneic models by specifically stimulating the slowly proliferative and nonproliferative cells into actively proliferating cells.

We then evaluated IL-15SA's effects on anti-tumor activity against murine mastocytoma (P815) and murine B cell lymphoma (A20). IL-15SA enhanced graft-versus-tumor (GVT) activity in these tumors following T cell infusion. Interestingly, IL-15 SA administration provided GVT activity against A20 lymphoma cells in the murine donor leukocyte infusion (DLI) model without increasing graft versus host disease. In conclusion, IL-15SA could be a highly potent T- cell lymphoid growth factor and novel immunotherapeutic agent to complement stem cell transplantation and adoptive immunotherapy.

## INTRODUCTION

IL-15 is a pleiotropic cytokine, which plays various roles in the innate and adaptive immune systems, including the development, activation, homing and survival of immune effector cells, especially NK, NK-T and CD8^+^ T cells [1]. IL-15, a member of the common gamma chain (γc) cytokine family, binds to a receptor complex that consists of IL-15Ra, IL-2Rβ and the γc chain [2, 3]. Furthermore, IL-15 functions as a key regulator in the development, homeostasis and activity of NK cells [4, 5]. IL-15 administration to normal mice or overexpression of IL-15 in a transgenic mouse model increases the number and percentage of NK cells in the spleen [6, 7], the proliferation and survival of NK cells, as well as their cytolytic activity and cytokine secretion. Our laboratory and others have previously shown that IL-15 administration can increase the NK cell number and function in recipients of stem cell transplantation [8–11].

The primary limitations in clinical development of recombinant human IL-15 (rhIL-15) are low production yields in standard mammalian cell expression systems and a short serum half-life [12, 13]. Moreover, the formation of the IL-15:IL-15Rα complex, with both proteins co-expressed in the same cell, can stimulate immune effector cells bearing the IL-2βγc receptor through a trans-presentation mechanism. The studies have shown that IL-15/IL-15Rα complex increases the affinity of the IL-15 to the IL-2Rβ approximately 150-fold, when compared with free IL-15 [14]. Various, types of complexes between IL-15 and soluble IL-15Rα have been generated that exhibit an enhanced biological activity and increased anti-tumor responses in animal models [15–17]

A superagonist mutant of IL-15 (IL-15N72D), which has increased Rβγc binding ability (4-5 fold higher than native IL-15) has been identified for therapeutic usages [18].

Based on these findings, the strong interaction of IL-15N72D and soluble IL-15Rα was exploited to create a new IL-15 superagonist complex with the IL-15N72D bound to the IL-15RαSu/Fc. The soluble fusion protein, IL-15RαSu/Fc, was created by linking the human IL-15RαSu domain with human IgG1 containing the Fc domain. Studies on IL-15:IL-15Rα complexes show an advantage of increased intracellular stability of IL-15 [19, 20]. Co-expression of both the IL-15N72D and IL-15RαSu/Fc proteins resulted in a soluble and stable complex with significantly longer serum half-life and increased biological activity, compared to native IL-15 [21], which was consistent with the results previously shown with other IL-15/IL-15Rα complexes [15–17, 22, 23]. As indicated above, this IL-15N72D:IL-15RαSu/Fc complex (IL-15SA, or ALT-803) was found to be >10-fold more active than free IL-15 in promoting *in-vitro* proliferation of IL-15-dependent cells [18].

IL-15 SA was previously shown to have potent anti-tumor activity in syngeneic murine models of multiple myeloma [24]. Here we show the potent effects of IL-15 SA on immune reconstitution and graft-versus-tumor (GVT)/ graft versus leukemia (GVL) activity in recipients of allogeneic hematopoietic stem cell transplantation (HSCT) in murine models.

## RESULTS

### Effects of IL-15SA on immune cells following HSCT

We first evaluated the effects of IL-15SA in T-cell depleted murine BMT models. We used two different MHC-mismatched allotransplant models. We have extensively investigated enhancement of immune reconstitution in our previous studies by cytokines and growth factors [10, 25–28]. The early reconstitution requires minimum 2-3 weeks post-transplant. Therefore, we administered cytokines either between days 21 and day 28 or days 14-28. We aimed to cover the same period in this study with day 17 and 24 administration schedule.

**Figure 1:**
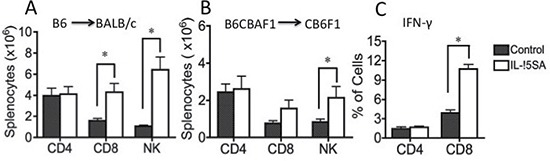
IL-15SA administration increases CD8^+^ T and NK cell numbers after transplantation (**A**) Lethally irradiated (11Gy) Balb/c recipients were transplanted with 5 × 10^6^ T-cell depleted (TCD) bone marrow (BM) cells from B6 mice. IL-15SA was administered via IP injection at 1 μg per mouse in two doses on days +17 and +24. Mice were sacrificed at day 28 after transplant, and spleens, thymi and BM were harvested. Single cell suspensions were prepared and stained with anti-H2Kd, -CD3, -CD4, -CD8, -Gr-1, -NK1.1, and -B220 antibodies, and analyzed with a flow cytometer. Each group contains 5 mice. Splenic numbers of CD4+ T, CD8+ T, and NK cells, are shown. *P < 0.05. Figure 1B and 1C. Lethally irradiated (12Gy) CB6F1 recipients were transplanted with 5 × 10^6^ T-cell depleted (TCD) bone marrow (BM) cells from B6CBA mice. IL-15 super agonist was administered via IP injection at 1 μg per mouse in two doses on days 17 and 24. Mice were sacrificed at day 28 after transplant, and spleens, thymi and BM were harvested. After preparation of single cell suspensions, cells were stained with anti-H2Kd, -CD4, -CD8 (**B**). Some splenocytes are also incubated as described for intracellular staining, then harvested and stained with anti-H2Kd, -CD4, -CD8 and IFN-γ antibodies and analyzed with a flow cytometer (**C**). Each group contains 5 mice. *P < 0.05

Lethally irradiated BALB/c recipients were transplanted with T cell depleted (TCD) bone marrow (BM) cells from B6 mice. IL-15SA was administered via intraperitoneal (i.p.) injection in two doses on days 17 and 24 after transplant. Animals were sacrificed on day 28. All recipients had more than 90% engraftment in the spleens and BMs. There was no significant difference in engraftment and cellularity in the spleens and BMs between IL-15SA and control groups (data not shown). Administration of IL-15SA significantly increased the number of CD8^+^ T and NK cells, whereas there was no change in CD4^+^ T cell numbers (Figure 1A). IL-15SA mostly increased CD8+ memory T cell population (CD44^high^) (data not shown). We observed similar activity in B6CBA^→^CB6F1 transplant model (Figure 1B), in which the animals were treated with the same dose and schedule. IL-15SA also augmented intracellular IFN-γ secretion by CD8^+^ but not CD4^+^ T cells in this model (Figure 1C).

**Figure 2:**
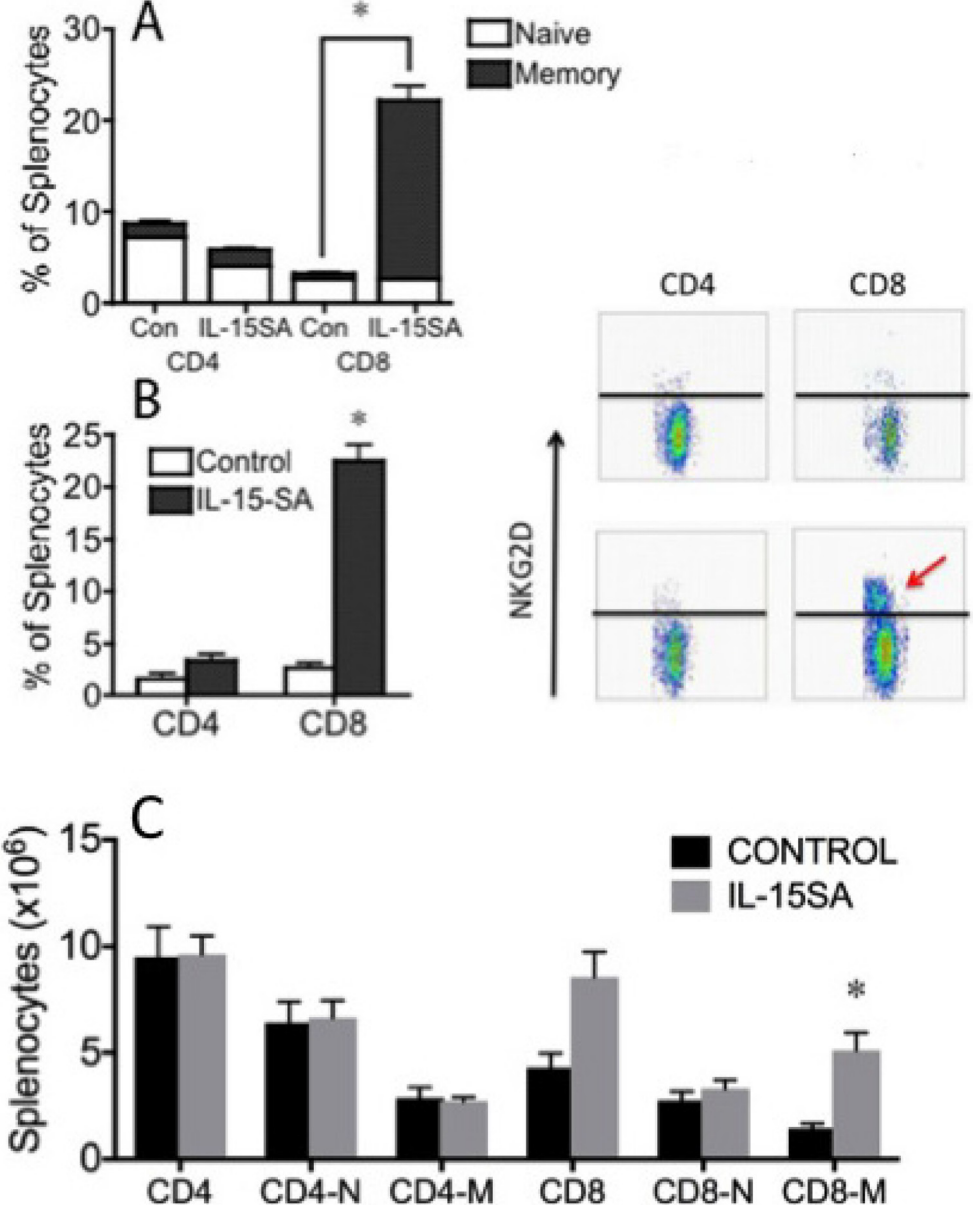
IL-15SA administration increases CD8^+^ CD44^+^ and CD8^+^NKG2D^+^ effector/memory T cells Lethally irradiated (12Gy) B6D2F1 recipients were transplanted with 5 × 10^6^ T-cell depleted (TCD) bone marrow (BM) cells from B6 mice. IL-15SA was administered via IP injection at 2.5 μg per mouse on days 28, 35 and 42. Mice were sacrificed at day 49 after transplant, and spleens were harvested. Single cell suspensions were prepared and stained with anti-H2Kd, -CD3, -CD4, -CD8, -CD44 and -NKG2D antibodies. Cells were acquired and analyzed with a flow cytometer (**A** and **B**). IL-15SA was administered via IP injection at 2.5 μg per mouse on days 35, 42 and 49. Mice were sacrificed at day 63 after transplant, and spleens were harvested. Single cell suspensions were prepared and stained with anti-H2Kd, -CD3, -CD4, -CD8, and -CD44 (**C**).

We then tested the effects of prolonged administration of IL-15SA on T cell reconstitution in an allogeneic transplant model. Again, recipients were treated with IL-15SA i.p. on days 28, 35 and 42 after MHC-mismatched HSCT (B6 ^®^ B6D2F1). We found that IL-15SA administration increased the CD8^+^ memory/effector T cell population, but did not show any activity on both CD4^+^ memory and naïve T cell populations. Interestingly, CD8^+^ naïve T cells also remained unaffected in both IL-15SA treated and untreated groups (Figure 2A). We also evaluated other activation markers on the lymphocytes. Interestingly, we found a 10-fold increase in NKG2D expression on CD8^+^ T cells, suggesting that some CD8^+^ T cells turn into effector/cytotoxic lymphocytes with innate-like phenotype (Figure 2B) after exposure to IL-15SA. These effects of IL-15SA on immune cells after HSCT are similar to previously observed changes in preclinical studies [29]. We did not find a significant change of surface CD107a expression on both CD4^+^ or CD8^+^ T cells, which is a marker of degranulation of the cytolytic perforin/granzyme pathway against the tumors (data not shown).

We then did another experiment to test the duration of IL-15 SA effects in our models. B6D2F1 recipients were lethally irradiated and transplanted with TCD B6 BM cells. The recipients were treated with IL-15 SA on days 35, 42 and day 49 and harvested on day 63 after allogeneic HSCT. observed similar spleen and CD4+T cell counts, but CD8+T cells T cells significantly increased two weeks after the last dose of IL-15 SA (Figure 2C). We concluded that the effects of IL-15 SA on immune reconstitution last at least two weeks after the administration.

**Figure 3:**
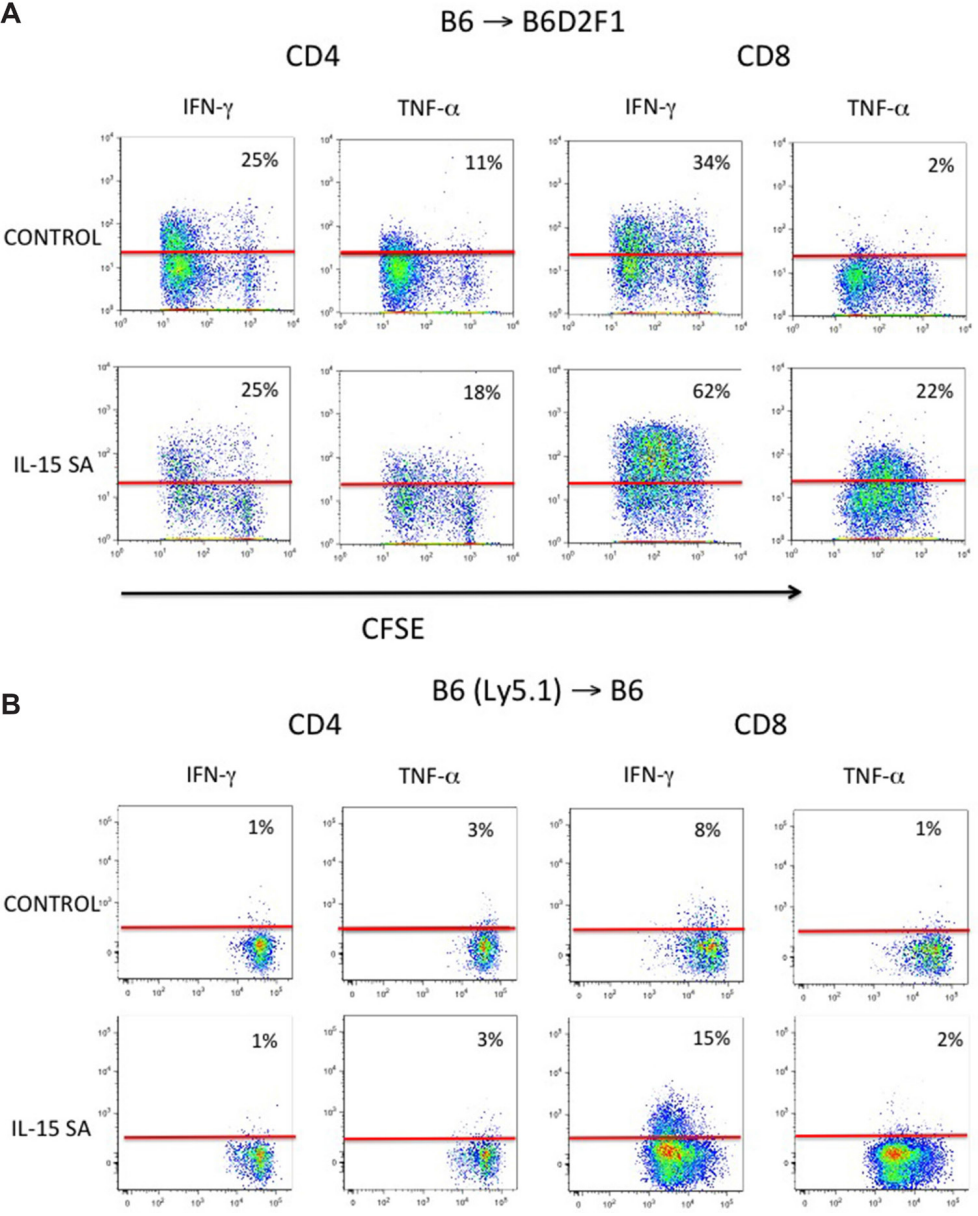
IL-15SA administration increases cytokine secretion and proliferation of CD8^+^ T cells in recipients of CFSE labeled T cells Lethally irradiated (1300 cGy) either B6D2F1 (**A**) or B6(Ly5.1) mice (**B**) were transplanted with CFSE labeled B6 splenocytes (30 × 10^6^) at day 0 and given either IL-15SA or a vehicle control (Day 0 post infusion). Mice were sacrificed on day 3 after CFSE labeled leukocyte infusion and splenocytes were stained with anti-CD4, -CD8, CD45.1 and -H2Kd antibodies. Cells then were analyzed by flow cytometry. Intracellular staining with anti-IFN-γ and anti-TNF-α antibodies after PMA and ionomycin stimulation was performed. Red line indicates boundary of isotypic control and arrows indicate increase in IFN-γ secretion in the slow proliferating CD8^+^ T cells.

We further examined the effects of IL-15SA on allogeneic proliferating cells in murine models. CFSE (carboxyfluorescein succinimidyl ester) labeled splenocytes from B6 mice were transferred into lethally irradiated B6D2F1 recipient mice. IL-15SA was given on the same day after splenocytes infusion. All animals were sacrificed three days after splenocyte infusion. IL-15SA treatment specifically promoted proliferation of slow-proliferating CD8^+^ T cells in conjunction with robust IFN-γ and TNF-α secretion, in allogeneic recipients of CFSE labeled-T-cell infusion. However, there was no effect of IL-15SA on CD4^+^ T cell proliferation (Figure 3A). Interestingly, we have not seen a significant increase in TNF-a secretion by CD8^+^ T cells following IL-15 administration in previous experiments [28], suggesting that IL-15SA is more potent than native IL-15 for inducing cytokine secretion in CD8^+^ T cells *in vivo*. We then evaluated IL-15SA activity in syngeneic recipients of CFSE labeled T-cell infusion. Again, we found that IL-15SA increased proliferation and IFN-γ secretion in adoptively transferred CD8^+^ T cells, but it did not increase their TNF-a secretion (Figure 3B). These results further suggest that additional stimulatory signals, such as TCR-MHC engagement in the allogeneic rather than the syngeneic adoptive T cell transfer setting, are potentially necessary to induce TNF-α secretion by IL-15SA stimulation.

### Antitumor activity of IL-15SA in murine tumor models

**Figure 4:**
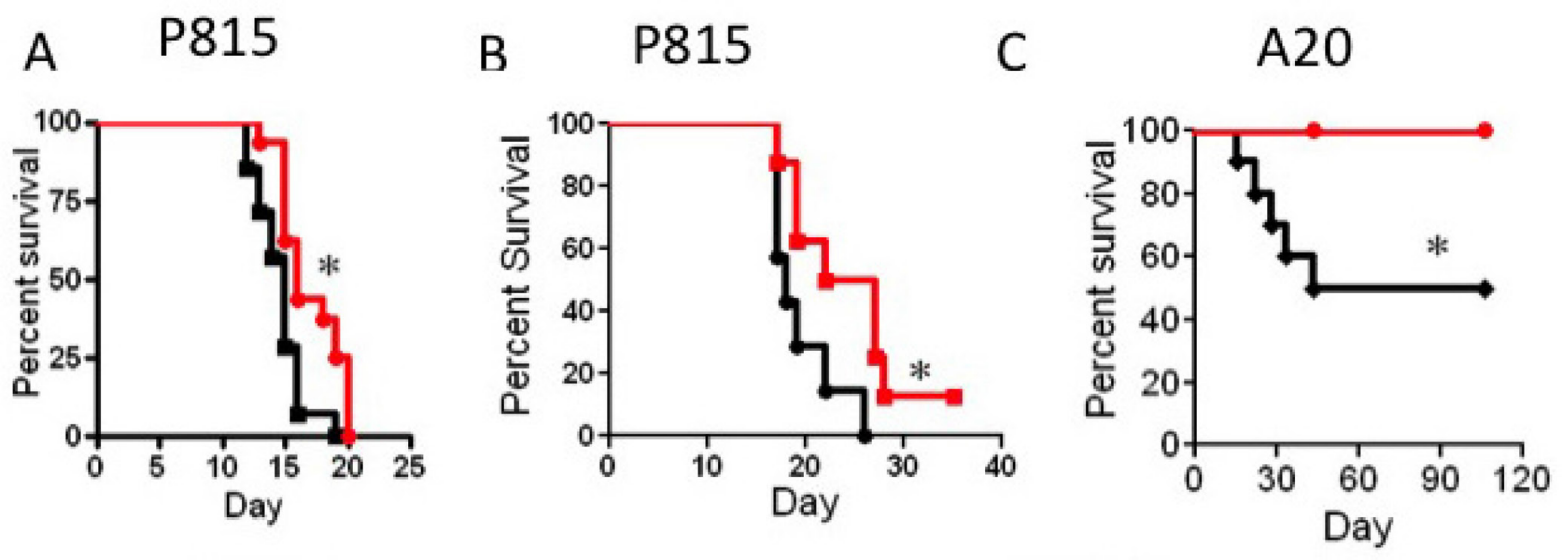
IL-15SA administration increases GVT activity after transplant (**A** and **B**) Lethally irradiated (13Gy) B6D2F1 recipients were transplanted with 5 × 10^6^ T-cell depleted (TCD) bone marrow (BM) cells from B6 mice. All recipients also received 1 × 10^4^ P815 cells on the day of transplantation along with 5 × 10^4^ (4A) or 1 × 10^5^ (4B) purified B6 T-cells. IL-15SA was administered via IP injection at 2.5 μg per mouse in two doses on days 7 and 14. Kaplan Mayer curves for this transplant modality are depicted as follows; vehicle control (black line) and IL-15 super agonist (red line). * = *p* < 0.05, and each group had 15 mice. (**C**) Lethally irradiated (13Gy) CB6F1 recipients were transplanted with 5 × 10^6^ T-cell depleted (TCD) bone marrow (BM) cells from B6 mice. All recipients also received 5 × 10^5^ A20 cells on the day of transplantation along with 1 × 10^5^ purified B6 T-cells. IL-15SA was administered via IP injection at 2.5 μg per mouse in two doses on days 7 and 14. Kaplan Mayer curves for this transplant modality are depicted as follows; vehicle control (black line) and IL-15 super agonist (red line). *=*p* < 0.05, and each group had 10 mice.

Next, we tested the anti-tumor activity of IL-15SA in two different tumor models; murine mastocytoma (P815) and murine B cell lymphoma (A20). First, we evaluated anti-tumor activity of IL-15SA in the P815 model, without T cell administration. We could not detect any significant graft-versus-tumor (GVT) activity in recipients of P815 in parent-F1 model when IL-15SA was administered without the T cell infusion (data not shown). When we infused a small amount of T cells into the B6^®^B6D2F1 model, we found that IL-15SA administration significantly enhanced anti-tumor activity against P815 tumor cells with two different T cell doses; 5 × 10^4^ and 1 × 10^5^ cells, respectively (Figure 4A and 4B). We did not observe any significant signs of GVHD in these experiments. Only two animals had a GVHD score of 1 because of minimal weight loss, which might be related tumor growth (in Figure 4A and no sign of GVHD in 4B). All animals died from tumor development with hind leg paralysis or presence of tumor metastasis in the autopsy. Therefore, IL-15SA administered with T cell infusion in the P815 model, provided a limited benefit to the tumor-bearing mice, compared to the control group.

In the A20 murine lymphoma model, we evaluated anti-lymphoma activity against A20 cells in recipients of allogeneic HSCT, with or without T cell infusion. A20 cells were kindly provided by Dr. Marcel van den Brink's laboratory (Memorial Sloan–Kettering Cancer Center, New York, NY), expressing triple gene construct with luciferase activity that allowed us to detect tumor growth with bioluminescence imaging (BLI). First, we developed an A20 murine tumor model and found that infusion of 1 × 10^5^ donor T cells provided a significant anti-tumor activity and survival benefit in the B6CBAF1 → CB6F1 (MHC-mismatched) model. Thus, we lethally irradiated CB6F1 mice and transplanted B6CBAF1 BM along with 1 × 10^5^ T cells. All animals received A20 tumor cells on the same day as the BM transplant. We found that all animals survived in the IL-15SA-treated group, which is statistically significant compared to the control group (Figure 4C).

**Figure 5:**
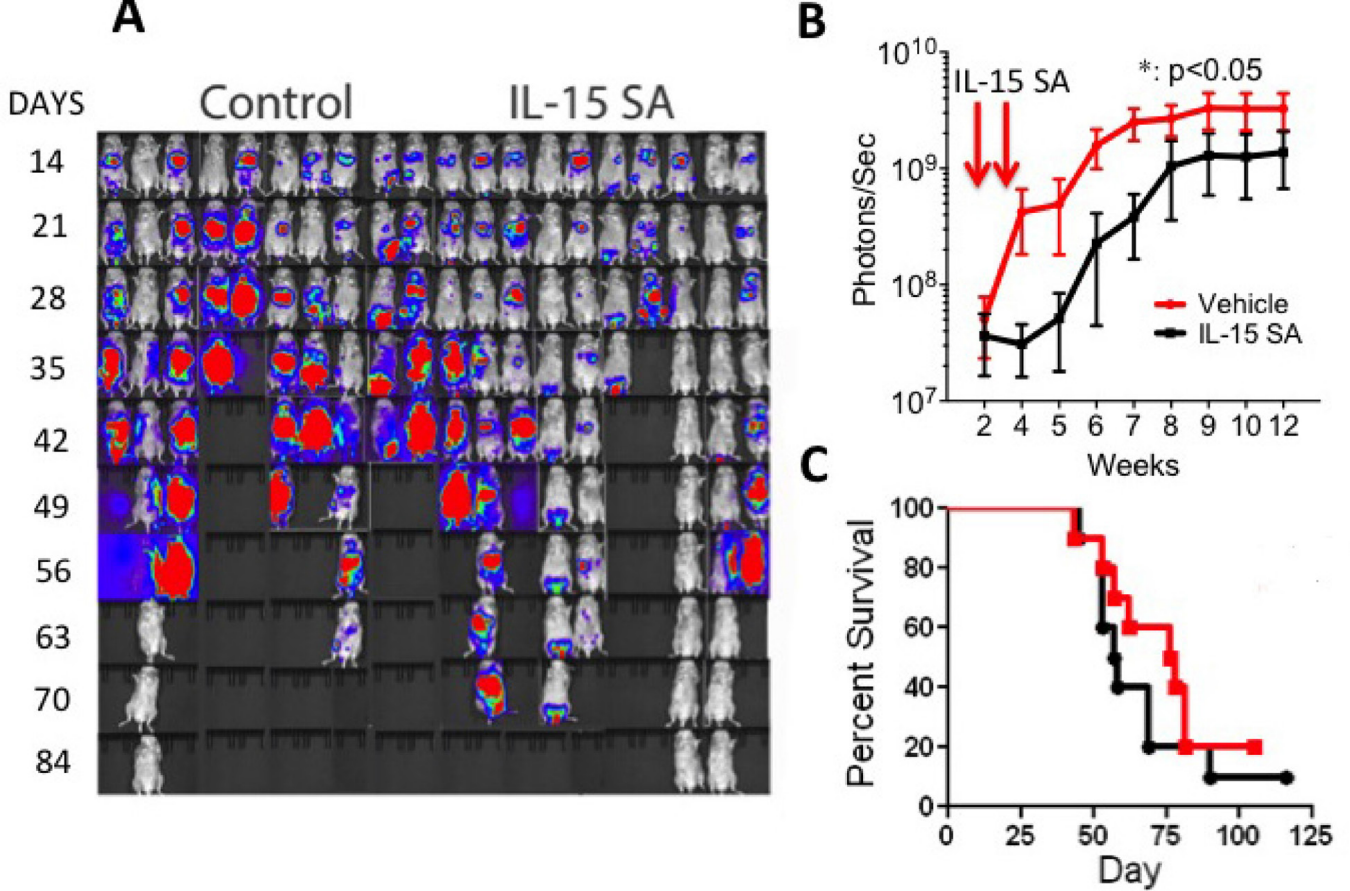
IL-15SA delays A20 lymphoma cells growth in recipients of HSCT Lethally irradiated (12Gy) CB6F1 recipients were transplanted with 5 × 10^6^ T-cell depleted (TCD) bone marrow (BM) cells from B6 mice. All recipients also received 5 × 10^5^ A20 cells on the day of transplantation along without T cell infusion. IL-15SA was administered via IP injection at 2.5 μg per mouse in two doses on days +7, and +14. *In vivo* luminescent imaging of experiment is shown in (**A**) Mice were injected with luciferin at 3.75mg per mouse, allowed to incubate for 8 mins, and then imaged for 3 mins. Control group on the left, IL-15SA on the right. Photon intensity is calculated and shown in (**B**) *= *p* < 0.05, and each group had 10 mice.

We then explored anti-lymphoma/leukemia activity of IL-15SA in allogeneic HSCT recipients, without any T cell infusion in the A20 model. Same dose and route of IL-15SA were used as previously described. Tumor growth was determined by intensity of photon measurements using IVIS bioluminescence system (Figure 5A, 5B). Although we observed that two administrations of IL-15SA could provide a delay of A20 cell growth *in vivo*, the delayed tumor growth by IL-15SA administration did not result in survival difference between the IL-15SA and the control group (Figure 5C). Interestingly, IL-15SA is still successful in generating anti-tumor activity against A20 lymphoma cells without a T cell infusion.

Taken together, the results of these experiments indicate that IL-15SA significantly increases the anti-lymphoma /leukemia activity in murine HSCT by effectively promoting the effector/memory CD8^+^ T and NK cell expansion and potently enhancing their effector functions.

### IL-15SA enhances anti-tumor activity with donor leukocyte infusion (DLI)

**Figure 6:**
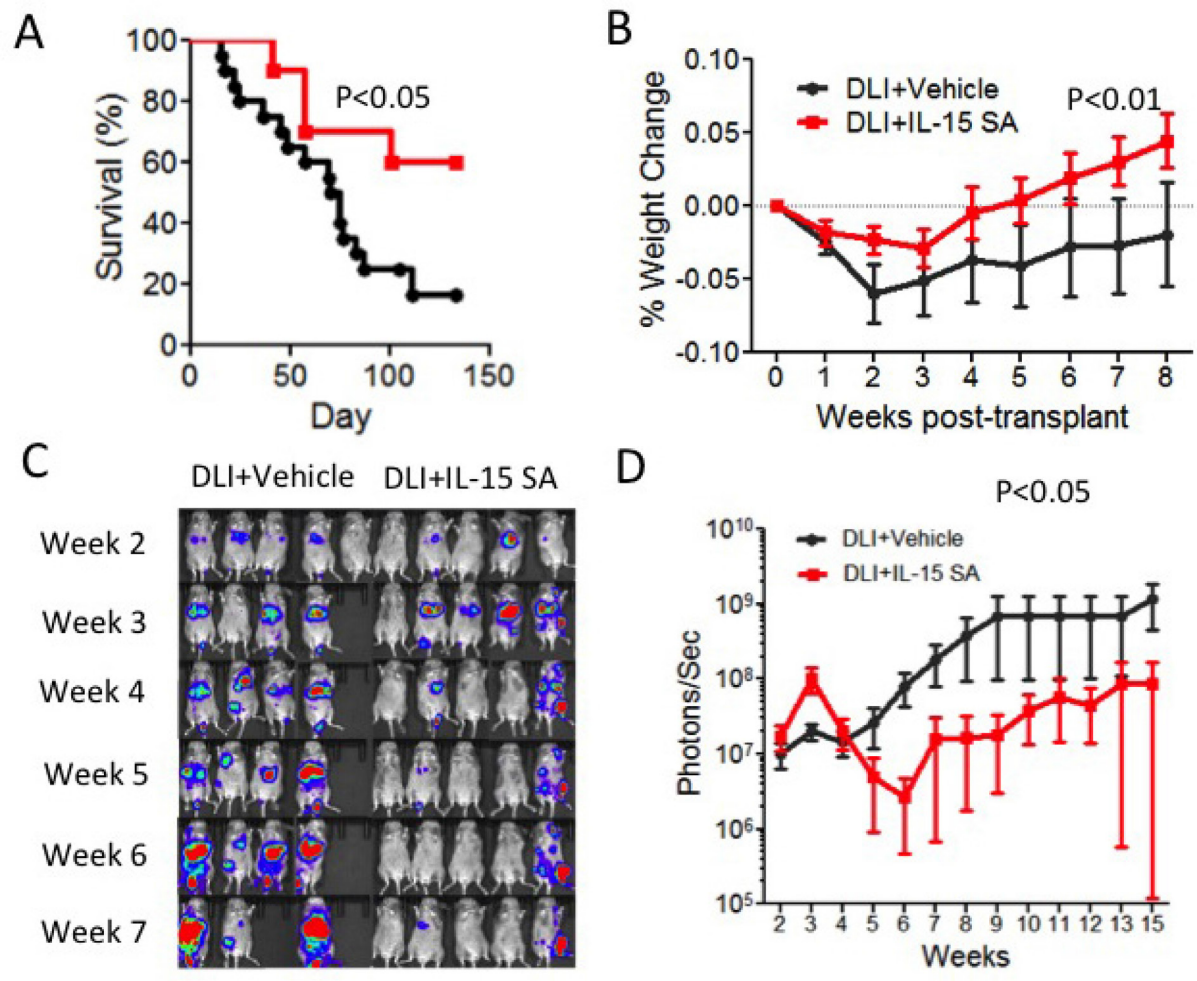
IL-15SA increases anti-tumor activity after DLI infusion in murine leukemia/lymphoma model Lethally irradiated (12Gy) CB6F1 recipients were transplanted with 5 × 10^6^ T-cell depleted (TCD) bone marrow (BM) cells from B6 mice. All recipients also received 5 × 10^5^ A20-TGL (H2K^d^) lymphoma cells with triple fusion gene carrying luciferase activity on the day of transplantation. The recipients of transplant received none or 2.5 × 10^5^ B6 T-cells isolated via CD5^+^ magnetic separation on day 14 after transplant. Animals received either intraperitoneal (IP) injections of IL-15 SA (1ug per mouse) or control on days 17 and 24 after transplant. Survival and weight curves of the groups are depicted as (**A** and **B)** (*N* = 10–20). Serial bioluminescence images are obtained by IVIS machine in varying time points as show. A20-TGL cells express Luciferase protein allowing *in vivo* bioluminescent imaging; mice were injected with 3.75mg Luciferin, incubated for 5 mins, and imaged for 3 mins. Serial bioluminescence images are obtained by IVIS machine in varying time points as show (**C**) representative of independent of two experiments). Control group on the left, IL-15 SA group on the right. Total flux (photons/sec) was measured for each mouse at each time point and plotted as a curve (**D**).

DLI has been developed as a strategy for management of relapse by increasing graft-versus-tumor effects after allogeneic HSCT [30]. DLI is used in nearly all malignant hematological diseases for which allogeneic HSCT is performed. However, the response to DLI varies with respect to the methods of cell collection, timing, cell dose infused, and even cell sub-type used (reviewed in [31]). To enhance the efficacy of DLI infusion would improve the outcome the patients who relapse after allogeneic HSCT. Therefore, we studied whether IL-15SA can be used to enhance the efficacy of DLI in animal models. To achieve this, we first developed a DLI model with recipients of allogeneic HSCT. Lethally irradiated CB6F1 recipients were transplanted with T-cell-depleted B6 BM cells. A20 murine lymphoma cells were infused at the day of transplant. Purified T cells were infused after tumor growth in recipients of allo HSCT. We found that a moderate dose of T cell infusion (2.5 × 10^5^ cells) provides GVL activity after tumor development in recipients of allo-HSCT (data not shown). The IL-15SA-treated group exhibited a better survival and less weight loss after transplant compared to the untreated group (Figure 6A and Figure 6B). We also monitored tumor growth in all animals with BLI. We found that IL-15SA administration resulted in significantly decreased photon intensity by BLI, which suggests IL-15SA was able to inhibit tumor growth (Figure 6C and 6D). 20% of recipients in the IL-15 SA group had score of 1, related to weight loss and mobility. No animals died from GVHD in the IL-15SA group. 30% animals had a low GVHD score between 1-2 in the control-DLI group. Only one animal died from GVHD in the control group. All other animals died from progression of leukemia/lymphoma (they have either a significant tumor growth or hind leg paralysis, which is a sign of tumor growth). We concluded that we did not observe an increase in GVHD score and weight loss in IL-15SA-treated group compared to the control group (Figure 6B) suggesting that IL-15SA did not promote GVHD in this murine HSCT model after a low dose T-cell infusion. Thus, IL-15SA administration after allogeneic HSCT enhances GVL/lymphoma activity without aggravating GVHD in the low-dose DLI murine models.

## DISCUSSION

We have previously shown that IL-15 enhances anti-tumor activity in recipients of allogeneic and haploidentical HSCT [10, 28]. IL-15 half-life is roughly 1 hour after administration and must be administered daily for treatment [15]. The long-term effects of recombinant human IL-15 (rhIL-15) have been studied in non-human primates, demonstrating that daily administration of IL-15 for 8-14 days resulted in lymphocytosis and leukocytosis, and white blood cell count returned to normal after discontinuation of IL-15 on day 28 in these studies [32]. Immunological parameters also returned to baseline on day 28 in the same studies. Due to the limitations with the short half-life and daily administration of IL-15, alternative dosing strategies of IL-15 require further assessment in the therapeutic setting.

Recently, Conlaon et al. reported that recombinant human interleukin-15 administration resulted in NK and CD8^+^ T cells redistribution, proliferation, activation of NK and CD8^+^ T cells and enhanced inflammatory cytokine production after daily bolus infusion [33]. Authors also mentioned that alternative dosing strategies have been studied to decrease the toxicity of cytokine. IL-15 SA (ALT-803) with a better safety profile and a longer half-life will potentially provide an advantage in clinical use.

We have shown in this study that a new IL-15 superagonist complex, IL-15SA, is a potent immunotherapeutic agent for stimulating NK and CD8^+^ T cells in recipients of allogeneic HSCT in murine models. IL-15SA promoted the expansion of CD8^+^ memory T cells and NK cells but not CD4^+^ T cells. IL-15SA also significantly increased the levels of NKG2D expression on CD8^+^ T cells. NKG2D, an activating receptor of innate immune cells, is mainly expressed on the surface of NK cells, γδ T cells, and activated CD8^+^ T cells. The NKG2D receptor plays a pivotal role in both innate and adaptive immunity against tumorigenesis and tumor surveillance [34, 35]. In the previous studies, IL-15SA induced memory CD8^+^ T cells to proliferate, upregulate receptors involved in innate immunity, secrete IFN-γ and acquire the ability to kill malignant cells in the absence of antigenic stimulation in murine models of multiple myeloma [24]

The studies described in this paper demonstrate for the first time that IL-15SA may have similar effects on the immune cells in the HSCT setting. Thus, it is conceivable that CD8^+^ T cells with high NKG2D expression (i.e., NKT cells) are induced by IL-15SA, and contributed to potent anti-A20 tumor activity in HSCT. This is consistent with the results from a recent report that showed NKG2D expression on CD8^+^ T cells is related to mediating GVHD and GVT by promoting the survival and cytotoxic function of CD8^+^ T cells [36]. NKG2D blockade was shown to attenuate GVHD, while allowing CD8^+^ T cells to regain anti-tumor activity. Besides functioning as an activating receptor for cell-mediated cytotoxicity of T-NK cells against tumors, NKG2D has also been suggested to act as a receptor to recruit T-NK cells to the tumor sites in which tumor cells overexpress stress-inducible NKG2D ligands [37].

In this study, we continue to observe the phenomenon of the IL-15SA not significantly promoting CD4^+^ T cell proliferation and activation. We have shown that IL-15 SA increased CD8+ T cell proliferation and IFN-g and TNF-a secretion from CD8+ T cells in recipients of allogeneic CFSE-labeled splenocyte infusion. Increased TNF-a secretion in allogeneic model might be related toan increase in T cells that may not be detectable in syngeneic model. These data may suggest that IL-15 SA may aggravate GVHD in allogeneic, though we have not seen this in our low dose T cell models Interestingly, IL-15SA increases IFN-g and TNF-a secretion from slow proliferative CD8 (+) T cells that may resemble the more homeostatic expansion of T cell in the lymphopenic environment [10, 25, 28]

We have previously shown that IL-15 administration after allogeneic HSCT may enhance the occurrence of GVHD in T cell-repleted models, but it does not have effect on GVHD after TCD-BMT [10]. Interestingly, IL-15 did not increase GVHD in recipients of a very low dose T cell infusion [28]. Using the same model in this study, we also found that IL-15SA did not increase the occurrence of GVHD and resulted in improved survival of haploidentical HSCT recipients of tumor bearing mice. Herein, we demonstrated that IL-15SA increased NK cell numbers in recipients of haploidentical HSCT. IL-15SA has also been shown to potently activate the cytotoxicity of NK cells [38]. NK cell alloreactivity in recipients of mismatched HSCT may suppress development of GVHD by decreasing host-derived antigen presenting cells [39]. Thus, it is conceivable that the increase of NK cell numbers and the enhancement of their cytotoxicity by IL-15SA administration in haploidentical HSCT not only contribute to the GVT but also decrease host-derived antigen presenting cells. The decrease in host-derived antigen presenting cells reduces the activation of host-specific CD8^+^ effector T cells which are responsible for GVHD. However, NK cell-associated GVT activity was apparently not strong enough to overcome the A20 tumor growth and improve the overall survival of the A20 lymphoma bearing recipient without T cell infusion pre- or post-transplant. Only a low dose of T cell infusion was shown to be required to provide survival advantage in the IL-15SA treatment group. This is likely the result of IL-15SA's unique capabilities of promoting the expansion of CD8^+^ memory T cells and enhancing their effector functions.

DLI has been developed as a strategy for relapse-management by increasing GVT effects after allogeneic HSCT [30]. DLI is used to treat most malignant hematologic diseases in recipients with relapsed disease after HSCT. The general response rate is less than 30% in patients with acute leukemia and is not durable [31]. Collins et al. has found the response rate to DLI to be less than 20% in acute leukemia patients [40]. In recent years, new methods have been developed to enhance the efficacy of DLI for the treatment of relapsed or persistent hematological malignancies after allogeneic HSCT [41]. Enhancing donor leukocyte activity with various cytokines has been explored. Interleukin-2 treatment after DLI in patients with relapsed leukemia after allo HSCT did not provide beneficial outcome, and increased the occurrence of GVHD [42]. Interleukin-15 has never been used following DLI in humans or murine transplant models.

The activity of DLI in murine models is affected by donor/host MHC Ag disparity, DLI dose, and tumor type. We developed the DLI model against the A20 murine lymphoma model system. In this model, we could detect a significant anti-tumor activity of DLI with a combination of IL-15 SA. We think that a major differences in our models and published data are T cell dose and the timing of tumor infusion. 2–5 × 10^7^ splenocytes were infused in the previous DLI studies in murine models [43–46]. T cell dose in those studies was approximately 1–2 log higher than the dose that was used in our studies (2.5 × 10^5^). As a result of that, the occurrence of GVHD diminished in this study. The timing of the tumor/leukemia cell infusion is also different in our study. We tried to mimic the clinical scenario for DLI infusion. The therapeutic DLI infusion is given after leukemia recurrence post-transplant in the patients with allogeneic HSCT. We aimed to test the efficacy of DLI after tumor development. Therefore, tumor cells were infused on the day of the transplant and the most of the recipients were detectable tumor by BLI at day 14 after HSCT. When we tested the timing of DLI, we observed that infusion at day 21 might be late to overcome the tumor development with a low dose T cell infusion (data not shown).

Our studies focused on exploring the activity of purified T cell-containing DLI and results revealed that IL-15SA administration significantly enhanced the activity of DLI in murine lymphoma model. Safety and feasibility of DLI and an IL-15SA combination would be further investigated in the future clinical studies. We have recently begun studies in non-transplant lymphoma models, and preliminary data reveals that IL-15SA can increase anti-lymphoma activity of autologous T cell infusion in normal mice after lymphodepletion, suggesting that IL-15SA may play a role in the lymphoma/leukemia therapy.

Herein, we have demonstrated that once a week administration of IL-15SA could provide sustained immunological activity and anti-tumor activity in murine tumor models, murine mastocytoma and murine B cell lymphoma. Substantial anti-tumor activity of IL-15SA has also been previously reported against multiple myeloma in syngeneic models [24]. This is likely due to the longer serum half-life of IL-15SA and its favorable pharmacokinetic profile compared to rhIL-15 [21]. The results of these studies support the weekly dosing regimen currently in various clinical trials for solid and hematological malignancies (Personal communication- Dr Wong, Altor Bioscience).

In summary, IL-15SA is a potent lymphoid growth factor and could be used as a powerful therapeutic for boosting the immune function in recipients of stem cell transplantation and adoptive T cell therapy without exacerbating GVHD. An evaluation of its clinical utility for stem cell transplantation and adoptive cellular therapies is warranted.

## MATERIALS AND METHODS

### Mice and bone marrow transplant (BMT)

Female C57BL/6 (B6, H-2K^b^), Balb/c (H-2K^d^), B6CBAF1 (H-2K^b/k^), CB6F1 (H-2K^b/d^) and B6D2F1 (H2K^b/d^) mice were obtained from the Jackson Laboratory (Bar Harbor, ME). Mice used in BMT experiments were between 10–-12 weeks of age. BMT protocols were approved by the Institutional Animal Care and Use Committee (IACUC) at Thomas Jefferson University.

Bone marrow (BM) cells were removed aseptically from femurs and tibias and T cells depleted (TCD) by incubation with anti-Thy 1.2 antibody for 30 min at 4°C, followed by incubation with Low-TOX-M rabbit complement (Cedarlane Laboratories, Hornby, Ontario, Canada) for 40 minutes at 37°C, or alternatively via anti-CD5 magnetic bead depletion (Miltenyi, Auburn, CA). Typical levels of contaminating T cells after complement depletion ranged from 0.2 to 0.5 percent of all bone marrow leukocytes.

Splenic T cells were obtained by positive selection with anti-CD5 antibodies conjugated to magnetic beads (Miltenyi, Auburn, CA). Cells (5×10^6^ BM cells with or without splenic T cells) were resuspended in Dulbecco Modified Eagle's Medium (DMEM) and transplanted by tail vein infusion (0.25 ml total volume) into lethally irradiated recipients on day 0. On day 0 prior to transplantation, recipients received 11 to 13 Gy total body irradiation (strain dependent) from a ^137^Cs source as a split dose with a 3 hour interval between doses to reduce gastrointestinal toxicity. Mice were housed in sterilized micro-isolator cages and received normal chow and autoclaved hyper-chlorinated drinking water (pH 3.0).

### Cell lines, antibodies, and cytokines

P-815 (H-2d) cell line was obtained from ATCC (Manassas, VA). A20 (H-2d) murine lymphoma cell line, retrovirally transduced to express a triple fusion protein consisting of Herpes simplex virus thymidine kinase, enhanced green fluorescent protein (eGFP) and firefly luciferase (TGL), was kindly provided by Dr. Marcel van den Brink (Memorial Sloan Kettering Cancer Center, New York, NY). Cells were cultured in RPMI with 10% FBS in atmosphere containing 5% CO2.

Anti–murine CD16/CD32 FcR block (2.4G2) and all of the following fluorochrome-labeled antibodies against murine antigens were obtained from BD Pharmingen (San Diego, CA): H2Kd (SF1-1.1), CD3 (500A2), CD4 (RM4-5), CD8 (53-6.7), CD25 (PC61), CD44 (IM7), CD45R/B220 (RA3-6B2), CD62L (MEL-14), NK1.1 (PK136), TNF-α (MP6-XT22), IFN-γ (XMG1.2), NK2GD, isotype controls; rat IgG2a-κ, rat IgG1-κ hamster, and IgG1-κ.

Cytokine complex IL-15SA (ALT-803) was kindly provided by Altor BioScience Corporation, Miramar, Florida. IL-15SA (ALT-803) was administered intraperitoneally, weekly at 1-2.5 μg/day.

### Flow cytometry

Single cell suspension of 10^6^ cells/25 μL was incubated at 4°C with CD16/CD32 FcR block. Subsequently, cells were incubated at 4°C with antibodies in a total volume of 50 μl. The stained cells were analyzed on a FACS Calibur flow cytometer (Becton Dickinson, San Jose, CA) with CellQuest software or LSRII cytometer (Becton Dickinson, San Jose, CA) with FlowJo software (Treestar, San Carlos, CA).

### Assessment of graft-versus-host-disease

The severity of GVHD was assessed with a clinical GVHD scoring system as previously described [47]. Briefly, ear-punched animals in coded cages were individually scored every week using 5 clinical parameters based on a scale from 0 to 2: weight loss, posture, mobility, fur, and skin. A clinical GVHD index was generated by summation of the 5 criteria scores (0–10). Survival was monitored daily. Animals with scores of at least 5 were considered moribund and were sacrificed.

### PMA-ionomycin stimulation and intracellular staining

Splenocytes were incubated with PMA (20 ng/mL) and ionomycin (1 μM) for 5 hours. Brefeldin A was added at a concentration of 10 μg/mL two hours following the addition of PMA and ionomycin. Cells were first stained with surface antibodies and then fixed and permeabilized with the BD Cytofix/Cytoperm Kit (BD Biosciences, San Diego, CA) and subsequently stained with intracellular antibodies.

### CFSE labeling

Cells were labeled with carboxyfluorescein succinimidyl ester (CFSE) as previously described [48]. Briefly, splenocytes were incubated with CFSE at a final concentration of 2.5 μM in PBS at 37°C for 20 minutes. Cells were then washed three times with PBS before intravenous injection.

### Statistics

All values shown in graphs represent the mean ± SEM. Survival data were analyzed using the Mantel-Cox log-rank test. For all other analysis, nonparametric unpaired Mann-Whitney-*U* test was used.
